# Integrating large language models for intuitive robot navigation

**DOI:** 10.3389/frobt.2025.1627937

**Published:** 2025-09-04

**Authors:** Ziheng Xue, Arturs Elksnis, Ning Wang

**Affiliations:** 1 Bristol Robotics Laboratory, University of Bristol, University of the West of England, Bristol, United Kingdom; 2 School of Computing and Digital Technologies, Sheffield Hallam University, Sheffield, United Kingdom

**Keywords:** home assistance robots, large language models, AI agent, LoRA fine-tuning, quantization

## Abstract

Home assistance robots face challenges in natural language interaction, object detection, and navigation, mainly when operating in resource-constrained home environments, which limits their practical deployment. In this study, we propose an AI agent framework based on Large Language Models (LLMs), which includes EnvNet, RoutePlanner, and AIBrain, to explore solutions for these issues. Utilizing quantized LLMs allows the system to operate on resource-limited devices while maintaining robust interaction capabilities. Our proposed method shows promising results in improving natural language understanding and navigation accuracy in home environments, also providing a valuable exploration for deploying home assistance robots.

## 1 Introduction

According to data from the United Nations, the global proportion of the population aged 65 and over has nearly doubled, rising from 5.5% in 1974 to 10.3% in 2024. It is predicted that between 2024 and 2074, this figure will double again, reaching 20.7% (Unfpa, 2024). The most pressing issue brought by population aging is the increasing strain it places on healthcare systems. While expanding nursing homes has been one solution, it often leads to challenges such as patient suffering and depression (Walker et al., 2007). Therefore, technological solutions increasingly focus on providing home care for the elderly, allowing individuals to live independently for longer periods. Despite significant technological advancements, there are still no commercially available robots capable of fully supporting elderly care at home. This is primarily due to the complexity of the tasks these robots must perform (Bodenhagen et al., 2019), the unpredictability of home environments (Alterovitz et al., 2016), and the need for unstructured interactions with users (Coronado et al., 2017).

LLMs (Zhao et al., 2023) represent a significant breakthrough in Natural Language Processing (NLP), enabling machines to perform a wide range of tasks, even those not explicitly included in their training data. Few-shot learning (Brown, 2020) and zero-shot learning (Wei et al., 2021) have demonstrated the power of LLMs to generalize across new tasks with minimal fine-tuning. However, LLMs’ significant capabilities are accompanied by equally huge computational and memory requirements, complicating their deployment in resource-limited settings or scenarios requiring high concurrency. Low-bit quantization has emerged as a critical technique for improving the efficiency and deployability of LLMs. Quantization reduces the bit-width of tensors, lowering memory and computation requirements while maintaining an acceptable level of performance. Thus, quantization is crucial for allowing LLMs to operate efficiently on resource-constrained devices, such as domestic robots, thereby broadening their practicality. In this paper, we demonstrate the deployment of a quantized LLM on a local robot, enabling effective operation on limited hardware.

As LLMs continue to develop, they are increasingly being integrated into the development of AI agents—intelligent entities capable of perceiving their environment, making decisions, and executing actions to achieve predefined goals (Analyticsvidhya, 2024). Recent developments in agent-based autonomous systems, like AutoGPT (Birr et al., 2024) and ChatGPT-plugins (Chatgpt plugins, 2024), have leveraged the power of large language models to generate text, control tools, and engage with users. These systems act as central controllers within artificial intelligence frameworks. Each agent is typically given a specific role, accompanied by tailored instructions and personality traits that shape how they interact with users. Researchers have found that shaping an agent’s “personality” can verifiably impact how LLMs perform in downstream tasks (Serapio-García et al., 2023).

However, despite the promise of AI agents, one of the critical challenges in deploying these systems is high deployment costs. Current AI agents rely heavily on large models, which are expensive to maintain through API calls and are challenging to scale for widespread commercial use. As a result, there is an increasing demand for smaller models that can deliver comparable capabilities at a reduced cost.

To address these challenges of cost and interpretability, we propose a novel AI agent architecture designed with modularity and hierarchical decision-making at its core. Our framework’s primary design objectives are: 1) To enhance system robustness under complex instructions by decoupling the core tasks of environmental understanding (EnvNet), high-level planning (AIBrain), and low-level navigation (RoutePlanner); and 2) To enable transparent traceability of the agent’s decision-making process for easier debugging and optimization. Our key contribution lies in demonstrating how this interpretable, three-part architecture not only functions efficiently on resource-constrained hardware via quantized LLMs but also provides a more robust and diagnosable solution for real-world home care scenarios compared to monolithic agent designs.

## 2 Related work

Home intelligent robots have become a key innovation in smart home environments, designed to assist users with various daily tasks. Recent research highlights their effectiveness in performing household chores, offering companionship, and enhancing security. For example, vacuum robots like iRobot’s Roomba are widely accepted for their efficiency in autonomous cleaning (Roomba, 2024). Companion robots have shown great potential in elderly care by reducing loneliness and providing emotional support. Additionally, security robots with surveillance capabilities enhance household safety through real-time monitoring. However, challenges remain in enabling these robots to navigate complex home environments and interact naturally with users. As research advances, future home intelligent robots are expected to offer greater autonomy and intelligence, tailoring services to user preferences and incorporating emotional computing to build stronger connections.

LLMs have made significant progress in natural language processing, demonstrating impressive generative abilities that can improve the functionality of intelligent robots. Models like GPT and T5 (Raffel et al., 2020) have advanced natural language understanding (NLU) and generation (NLG), enabling more complex interactions. Recent work has focused on combining LLMs with Vision-Language Pre-training (VLP) models (Dai et al., 2022), enhancing multimodal capabilities that support a range of applications, such as 3D comprehension and decision-making in autonomous systems (Rajvanshi et al., 2023; Elksnis et al., 2025). This combination is essential for improving the perception and interaction skills of home robots. Furthermore, the utility of local quantized LLMs in semantic navigation has been explored, indicating their potential for enhancing robotic navigation capabilities in complex environments (Elksnis et al., 2024).

AI Agents represent a key area of research, defined as autonomous systems capable of performing tasks and interacting with their environment. These systems can be categorized into various types, including conversational agents (Miao et al., 2023), task-oriented agents (Gur et al., 2023), and fully autonomous agents (Wang et al., 2023). They rely on advanced technologies such as natural language processing, machine learning, and knowledge graphs to improve their functionality. While models like GPT and BERT enable natural language interactions, challenges such as explainability and contextual understanding remain significant barriers to broader adoption (Shapira et al., 2023). Future advancements are expected to improve agent autonomy and multimodal capabilities, ultimately offering more integrated solutions across various sectors.

In contrast to existing works, our research contributes a framework that addresses not only the computational efficiency via quantized LLMs but also the critical architectural limitations of current AI agents. While many systems focus on integrating a single large model, our work emphasizes a principled, modular design consisting of EnvNet, AIBrain, and RoutePlanner. This hierarchical structure distinguishes our approach by providing clear interpretability at each stage of the decision-making process. It moves beyond simply using an LLM for language tasks and instead presents a blueprint for a more robust, diagnosable, and scalable agent architecture tailored for the complexities of home care robotics.

## 3 Proposal framework

### 3.1 System architecture overview

**FIGURE 1:**
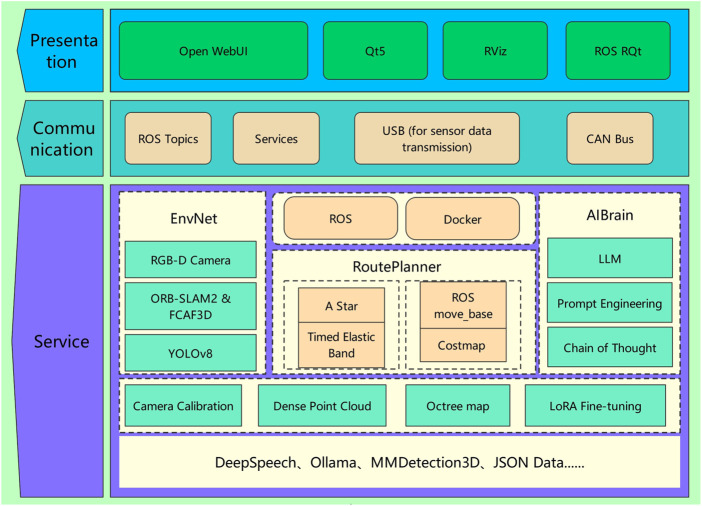
Intelligent home assistance robot system architecture.

The system architecture of the intelligent home assistance robot, as illustrated in Figure 1, emphasizes the coordination between the core hardware and software components. At the heart of the architecture is the modular design, enabling seamless integration and communication across all subsystems for real-time task execution. These core elements work together to ensure precise perception, flexible decision-making, and efficient navigation in dynamic environments. The details of this architecture are outlined below:

Our framework consists of three core components: EnvNet, which facilitates environmental perception; RoutePlanner, responsible for efficient path planning and obstacle avoidance; and AIBrain, which processes and interprets user instructions using LLMs. The AI agent enables the robot to navigate efficiently using a wheeled locomotion system, allowing it to move through obstacles and cluttered spaces designed for human environments while performing tasks at height. This approach aims to improve the robot’s capabilities in supporting daily activities, making it a valuable assistant in home care settings.

- EnvNet handles environment perception using an RGB-D camera. To reduce the computational load, the system is divided into two parts: fixed objects in the home, such as sofas and beds, are pre-detected using ORB-SLAM2 (Mur-Artal and Tardós, 2017) and FCAF3D (Rukhovich et al., 2022), with their positions, colors, and other attributes stored. Non-fixed objects, like fruits, are detected in real-time using YOLOv8 (Ultralytics, 2023).
- RoutePlanner ensures stable and efficient movement through complex environments using a wheeled base. The robot employs omnidirectional wheels, allowing it to move in any direction without adjusting its centre of mass. This wheeled path planning system enables the robot to perform tasks requiring precise movement and positioning, such as easily navigating through narrow spaces or avoiding obstacles.
- AIBrain interprets complex natural language commands and executes intelligent decisions. This component is designed to imitate human thinking, allowing the AI agent to understand commands such as “I want to eat an apple,” go to the kitchen, recognize the apple, and then bring it to the user.

**FIGURE 2:**
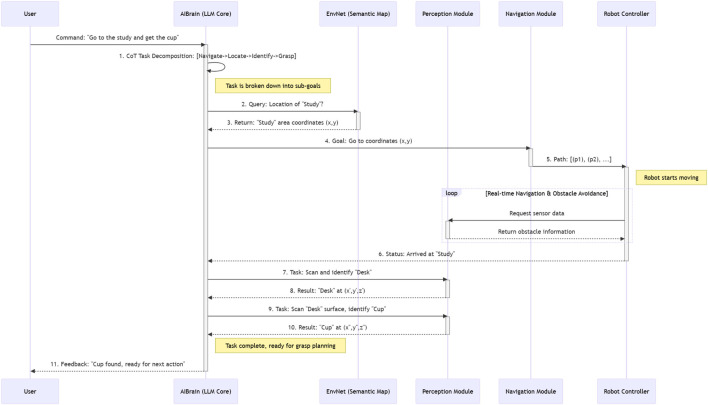
Swimlane diagram illustrating the operational workflow of our proposed AI agent framework. The process starts with a user command and details the sequential interaction between the AIBrain, EnvNet, and RoutePlanner modules to achieve the final robot action. This visualization highlights the system’s modularity and hierarchical decision-making process.

To further clarify how these modules collaborate to execute a user’s command, the operational workflow is detailed in Figure 2.

#### 3.1.1 Hardware components

The experimental platform is constructed from several key hardware components, detailed in Table 1. The most critical of these are outlined below:

- Jetson Orin NX: The Jetson Orin NX serves as the primary processing unit, equipped with NVIDIA’s powerful AI computing architecture. It handles the demanding AI inference and real-time processing tasks, ensuring efficient execution of deep learning models, particularly for object detection and SLAM algorithms. Its low power consumption and high performance make it suitable for the embedded nature of the robot.
- Mecanum Wheels: The Mecanum wheels enable the robot to navigate omnidirectionally, providing it with enhanced mobility in constrained environments. This capability is particularly crucial when navigating in tight or cluttered spaces, allowing the robot to move laterally and rotate in place, ensuring precise path adjustments during navigation tasks.
- RGB-D Camera: The RGB-D camera captures both color and depth information from the environment. It plays a vital role in the perception system (EnvNet) by generating real-time 3D maps of the surroundings. This data is crucial for environmental understanding, object detection, and SLAM, allowing the robot to localize itself accurately and recognize both fixed and non-fixed objects.

**TABLE 1:** Hardware Configuration.

Component	Parameter	Value
Robot	Dims (LWH)	266 × 230 × 402 mm
	Drive	4 × MG513 DC Motors w/Swing Suspension
	Wheels	4 × 75 mm Mecanum
	Controller	STM32F407VET6
	Encoders	500-line GMR
	Battery	12V/2.6Ah Li-ion
Compute	Main Comp.	NVIDIA Jetson Orin NX (16 GB)
	CPU	8-core Arm Cortex-A78AE
	GPU	1024-core Ampere
	RAM	16 GB LPDDR5
	AI Perf.	Up to 100 TOPS
Sensing	RGB-D Cam	Orbbec Gemini 2 Pro
	IMU	MPU-9250 (9-DOF)
Manipulation	Arm	6-DOF Robotic Arm
	Servos	S20F High-Torque Digital Servos
	Payload	200–300 g
	Weight	1.2 kg
	Controller	C06B w/Built-in Kinematics
Software	OS	Ubuntu 20.04 with ROS 1 Noetic

#### 3.1.2 Software components

- User Interface (UI) Module: The UI Module is developed using Qt5, offering users a graphical interface to interact with the robot. It supports inputting natural language commands, making the robot more user-friendly. The intuitive design enhances user experience and allows for smooth communication with the robot by displaying feedback and requests for clarification when needed.
- Instruction Processing Module: Deploy the LLMs on the Ollama platform; the Instruction Processing Module interprets natural language commands from the UI Module. This module converts complex user requests into actionable commands for the robot to perform tasks. The LLMs ensure accurate command interpretation, with mechanisms for handling ambiguities by interacting with the user for further clarifications.
- SLAM Module: The system uses ORB-SLAM2 to generate real-time sparse point cloud maps of the environment. These sparse maps are processed further into dense point clouds, which are stored using an octree structure. This allows for efficient mapping of the robot’s surroundings, enhancing both the robot’s localization capabilities and the precision of its path planning.
- Object Recognition Module: The object recognition system employs a hybrid approach, combining FCAF3D and YOLOv8 algorithms. FCAF3D is utilized for detecting and classifying fixed objects, such as furniture, in 3D space, ensuring high precision. YOLOv8, on the other hand, is applied to recognize and classify non-fixed objects, such as fruits, in real-time. This combination allows the robot to operate effectively in dynamic home environments.
- Navigation & Control Module: The move_base package from ROS is used for navigation and real-time control. It integrates the global and local path planning functionalities, relying on data from both the SLAM and Object Recognition modules. This module ensures that the robot can navigate complex environments while avoiding obstacles and adjusting its path in real-time to complete assigned tasks efficiently.

### 3.2 Perception module (EnvNet)

This module constitutes the foundation of the robot’s environmental awareness, enabling it to construct precise 3D maps, identify both fixed and non-fixed objects. EnvNet integrates ORB-SLAM2 for mapping with FCAF3D (Rukhovich et al., 2022) and YOLOv8 for object detection, enabling the robot to remain cognizant of environmental changes, which is essential for the AI agent’s overall performance.

#### 3.2.1 SLAM implementation

- ORB-SLAM2: ORB-SLAM2 is a feature-based visual SLAM system that performs real-time localization and mapping of the environment. It utilizes the ORB feature point algorithm to extract key features from camera images, enabling accurate position tracking and 3D map generation. ORB-SLAM2 generates sparse point clouds in real time. These point clouds are then processed to create dense point clouds, while an octree structure is used to efficiently store and manage the 3D data. This allows the robot to continuously update its environmental map with high precision. The dense point clouds provide detailed descriptions of the surroundings, which are essential for navigation and interaction with objects in the home.

#### 3.2.2 Object recognition

- Fixed Objects: For detecting and recognizing fixed objects such as furniture, FCAF3D is employed as the primary 3D object detection method. FCAF3D excels in handling complex indoor 3D point cloud data without relying on predefined geometric assumptions. The architecture consists of a backbone, neck, and head design that ensures efficient multi-scale feature extraction and high-precision detection. The loss function of FCAF3D incorporates classification loss, regression loss, and centerness loss, which together optimize the prediction of object classes, positions, and 3D bounding boxes.
- Non-Fixed Objects: YOLOv8 is used for the real-time detection and classification of non-fixed objects, such as fruits or other items that may change location over time. The model is capable of performing object detection and classification at high speeds, which is essential for a robot operating in dynamic environments like a home. YOLOv8 ensures that the robot can quickly identify and respond to moving objects, enabling it to perform tasks such as picking up or interacting with items as they are encountered.

### 3.3 Navigation and control module (RoutePlanner)

The RoutePlanner module is a crucial component of the AI agent, responsible for handling path planning and movement control. It ensures the robot can navigate through complex indoor environments, avoiding obstacles, and following the most efficient routes to complete tasks. By integrating both global path planning through the A* algorithm and local path optimization using ROS’s move_base package, RoutePlanner enables seamless navigation even in dynamic and cluttered environments. This module plays a key role in determining the robot’s real-time responses to environmental changes and obstacle detection, making it essential for the overall functionality of the AI agent.

#### 3.3.1 Global path planning

- 2D Grid Map: The 2D grid map is used to represent the environment, marking areas as either free, occupied, or unknown. It provides the necessary information for global path planning algorithms to select a viable path.
- A* Algorithm: The A* algorithm calculates the optimal global path based on the 2D grid map. It uses a heuristic function to determine the shortest path from the start to the goal:

$$f\left(n\right)=g\left(n\right)+h\left(n\right)$$

where g(n) represents the actual cost from the start to the current node, and h(n) is the estimated cost from the current node to the goal. This ensures that the robot finds the most efficient route through the known environment.

#### 3.3.2 Local path optimization

- ROS move_base: The move_base package in ROS handles local path planning and dynamic obstacle avoidance. It uses the local costmap generated from the robot’s sensors to navigate around obstacles in real time, ensuring the robot can reach its destination even in changing environments.
- Layered Costmap: The costmap is divided into layers: the static map layer provides pre-existing knowledge of obstacles, while the obstacle layer updates dynamically based on sensor input. The inflation layer expands obstacle areas to ensure a safety buffer for the robot.

### 3.4 Decision-making module (AIBrain)

#### 3.4.1 Motivation and proposal

- Overview of AIBrain: The AIBrain module plays a pivotal role in the functionality of the AI agent, facilitating the processing of natural language commands and enabling intelligent decision-making for complex tasks. This module harnesses the power of large language models (LLMs) to seamlessly bridge the gap between human directives and robotic execution, thereby enhancing the overall efficiency and effectiveness of the AI system.
- Importance of LLMs: Large language models are integrated into the AIBrain module to interpret and process natural language commands with high accuracy. Advanced techniques, such as prompt engineering and Chain-of-Thought reasoning, are employed to augment the robot’s understanding and response to complex tasks. These techniques enable the AI agent to better comprehend and execute multi-step instructions, leading to improved performance in various scenarios.
- Need for Fine-tuning and Quantization: Fine-tuning is a crucial step in adapting LLMs to specific tasks, ensuring that the model can accurately understand and respond to task-related commands. Additionally, quantization is essential for efficient deployment of the model on resource-limited hardware, as it reduces the memory footprint and computational overhead, thereby enabling real-time decision-making in constrained environments.

#### 3.4.2 Technical implementation

- Prompt Engineering: In this study, using prompts is crucial in producing semantic information from LLMs for semantic navigation tasks. Specifically, two main types of prompts are designed: room classification prompts and goal selection prompts. For room classification, the LLM is queried with a list of objects observed at a specific location and asked to determine the room type based on these objects. For goal selection, the LLM is provided with a free-text description of the target item to be retrieved and asked to suggest the most likely room and object location within that room where the item can be found. These prompts are carefully constructed to align with the capabilities of the LLMs and facilitate the extraction of relevant semantic knowledge required for navigation decisions. To ensure full reproducibility, the complete templates for these prompts are provided in Supplementary Appendix SA.
- Chain-of-Thought: The Chain-of-Thought (CoT) prompting technique is employed to enhance the reliability and interpretability of LLM responses. This technique requires the LLM to generate a justification for its answers and the final answer itself. By encouraging the LLM to express its reasoning process, the CoT prompting technique helps to produce more consistent and explainable responses. In our experiments, the LLMs are prompted to provide a chain of thought that explains their room classification and goal selection decisions. While this approach sometimes results in varied formatting of the responses, it generally leads to more reliable and reasonable navigation decisions. The use of CoT prompting underscores the importance of transparent and interpretable AI systems, particularly in safety-critical applications such as semantic navigation.
- LoRA (Low-Rank Adaptation): LoRA is a fine-tuning technique designed to adapt large pre-trained language models to specific tasks, while significantly reducing the number of trainable parameters. LoRA introduces a bypass that performs dimensionality reduction and expansion instead of training all parameters. Specifically, two matrices, A and B , are introduced where matrix A is randomly initialized, and matrix B is set to zero. The main model’s parameters remain frozen during training, while only matrices A and B are updated. The modified weight matrix is expressed as $W_{0}+\Delta W=W_{0}+BA$ , where $B\in R^{d\times r}$ , $A\in R^{r\times k}$ , and r≪min(d,k) .
- Quantization for Local Deployment: To minimize the memory footprint and reduce the computational overhead for running the large language model on resource-limited hardware, low-bit quantization techniques (such as 8-bit or lower) are applied. This process compresses the model weights, enabling efficient deployment on devices like the Jetson Orin NX without compromising the robot’s decision-making abilities.

### 3.5 Implementation details

To ensure the reproducibility of our work, we detail the key hyperparameters for the core components of our system. These parameters were optimized for performance and efficiency on our target hardware platform. The complete configuration is presented in Table 2.

**TABLE 2:** Key component hyperparameter Configuration.

Component	Parameter	Value
ORB-SLAM2	Number of Features	2000
	Scale Factor	1.2
	Pyramid Levels	8
	Depth Threshold	40 mm
FCAF3D	Voxel Size	0.02 m
	Training Epochs	50
	Learning Rate	1e-4
YOLOv8	Model Variant	YOLOv8n
	Pretrained Weights	COCO
	Input Size	640x640
	Confidence Threshold	0.25
	IoU Threshold (NMS)	0.45
LoRA Fine-tuning	Rank (r)	16
	Alpha (α)	32
	LoRA Dropout	0.05
	Training Epochs	3
	Batch Size	4
	Learning Rate	2e-5
Model Quantization	Bit Precision	4-bit (NF4)

## 4 Experimental setup

The experiments presented in this study are designed as a proof-of-concept to validate the core efficacy of our proposed modular framework in representative home scenarios. We acknowledge that the diversity of environmental conditions and the scale of the command dataset require further exploration, which we plan to address in future work. Nevertheless, we contend that the current results, though preliminary, demonstrate the fundamental feasibility and potential of our architectural design.

### 4.1 Experimental environment

Experiments were conducted in real-world home environments, selecting various rooms including the living room, bedroom, kitchen, bathroom, and study room. Each room was furnished with typical household objects such as chairs, tables, beds, refrigerators, and bookshelves to simulate actual living conditions. These fixed objects were used as landmarks for navigation and room type inference tasks.

### 4.2 EnvNet experimental phases

#### 4.2.1 SLAM system and mapping generation

**FIGURE 3:**
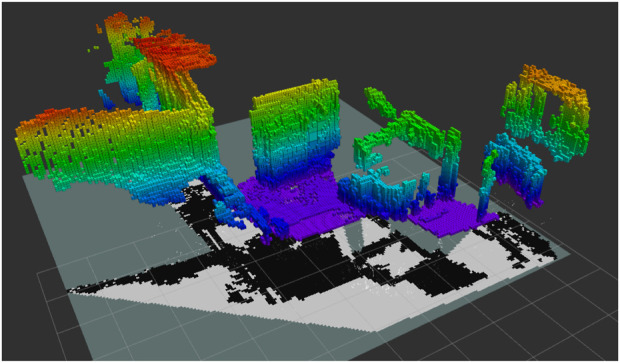
OctoMap of indoor rooms.

**FIGURE 4:**
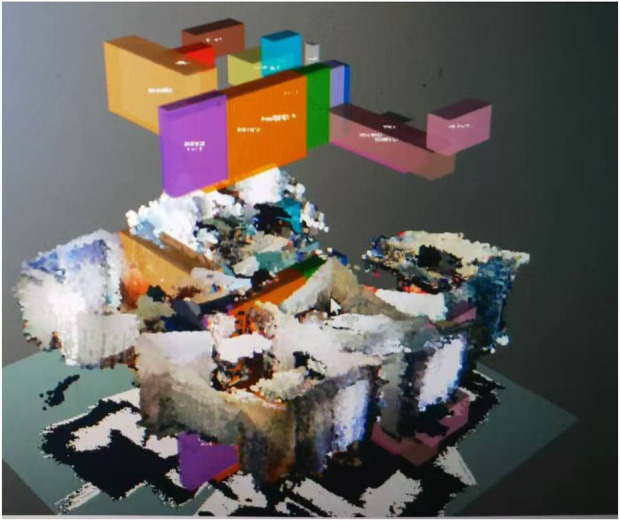
FCAF3D detecting the entire household room.

- 3D Map Generation: Utilizing the optimized ORB-SLAM2 system, a 3D OctoMap of the indoor environment was created (Figure 3). The map includes detailed spatial information and allowed the robot to navigate complex household layouts.
- SLAM + FCAF3D: The purpose of this part was to evaluate the accuracy of FCAF3D in detecting and classifying objects in an indoor environment. The robot initially conducted a thorough scan using the ORB-SLAM2 algorithm to build a 3D map of the environment, as illustrated in Figure 4. This map provided a spatial understanding of the room. The FCAF3D model was employed to detect and classify various objects, including bed, bookshelf, cabinet, chair, curtain, desk, door, garbage bin, refrigerator, sink, sofa, table, toilet, and window. To ensure consistent testing conditions, all scans were conducted at night with all room lights turned on, to avoid variability due to daylight. Each room was scanned four times to assess the model’s consistency.
  - Independent Variable: The type of object in the indoor environment.
  - Dependent Variable: The accuracy of object detection and classification by the FCAF3D model.
  - Controlled Variables: Testing conditions such as lighting (performed at night with all lights on), the number of scans (four scans per room), and the indoor environment being consistent across trials.

### 4.3 AIBrain experimental phases

#### 4.3.1 Fine-tuning LLMs using generated data

**FIGURE 5:**
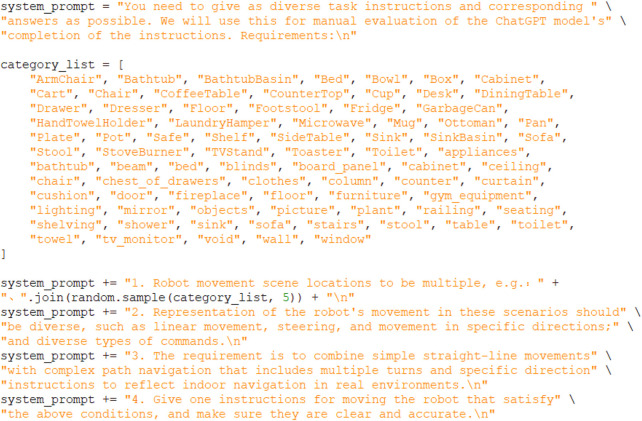
LLMs fine-tuning data generation code example.

During the fine-tuning of LLMs, an experiment was conducted to address the lack of specialized datasets, which made direct fine-tuning of the large language model challenging. To solve this problem, GPT’s API was used to generate 10,000 training samples through prompt engineering (Figure 5). These generated samples provided a suitable dataset for the subsequent fine-tuning process.

**FIGURE 6:**
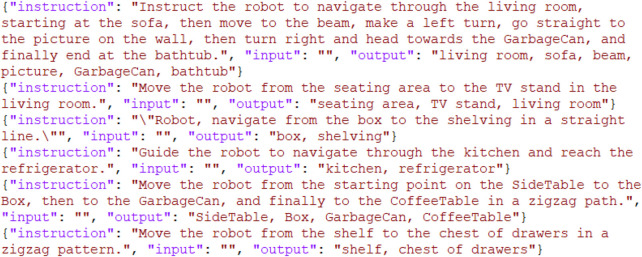
LLMs fine-tuning data sample.

After generating the dataset (Figure 6), LoRA (Low-Rank Adaptation) was applied for efficient fine-tuning of the model. The experimental setup to evaluate the effectiveness of LoRA involved:

- Independent Variable: The use of LoRA for fine-tuning the LLM.
- Dependent Variable: Performance metrics, specifically training loss and response quality.

#### 4.3.2 LLM performance evaluation

To systematically evaluate the performance of various LLMs, including Gemma, Mistral, Llama 3, and a fine-tuned Llama 2, we conducted an experiment designed with clear variables:

- Independent Variable: The LLM being evaluated (Gemma, Mistral, Llama 3, fine-tuned Llama 2).
- Dependent Variables:
  – Accuracy: The ability of the model to correctly extract the intended target from a given command (e.g., extracting “couch” from “Navigate to the couch”).
  – Response Time: The time taken by the model to respond to the given prompt.
•Controlled Variables: Testing conditions, including the format of the prompts, computational environment, hardware specifications, and the test set.

The test set consisted of both simple and complex commands to assess the models’ ability to comprehend and execute instructions with different levels of complexity:

- Simple Commands: For example, “Navigate to the couch,” which involves identifying a single target destination.
- Complex Commands: For example, “Instruct the robot to navigate through the living room, starting at the sofa, then move to the beam, make a left turn, go straight to the picture on the wall, then turn right and head towards the GarbageCan, and finally end at the bathtub.” These commands involve identifying multiple sequential navigation targets.

The purpose of this experiment was to determine the performance differences among various LLMs in terms of accuracy and efficiency when processing navigation commands. By evaluating both accuracy and response time for the same set of ten simple and ten complex commands, we aimed to identify which LLM demonstrated superior performance under different command complexities.

#### 4.3.3 Room type inference

To evaluate the performance of the combined SLAM and FCAF3D system, and subsequently infer room types using LLMs.

- Room Type Inference with LLM: Following object detection, this part of the experiment aimed to determine the accuracy of inferring the room type based on detected objects. The detected objects were used as input to various LLMs to classify the room as one of the predefined types: bathroom, bedroom, kitchen, living room, or study. Each room type was associated with specific combinations of objects typically found in that room.
  - Independent Variable: Different LLMs used for room type inference.
  - Dependent Variable: The accuracy of the room type classification.
  - Controlled Variables: The set of detected objects (output from FCAF3D), the prompt format provided to the LLMs, and the computational environment in which the LLMs were tested.

**FIGURE 7:**
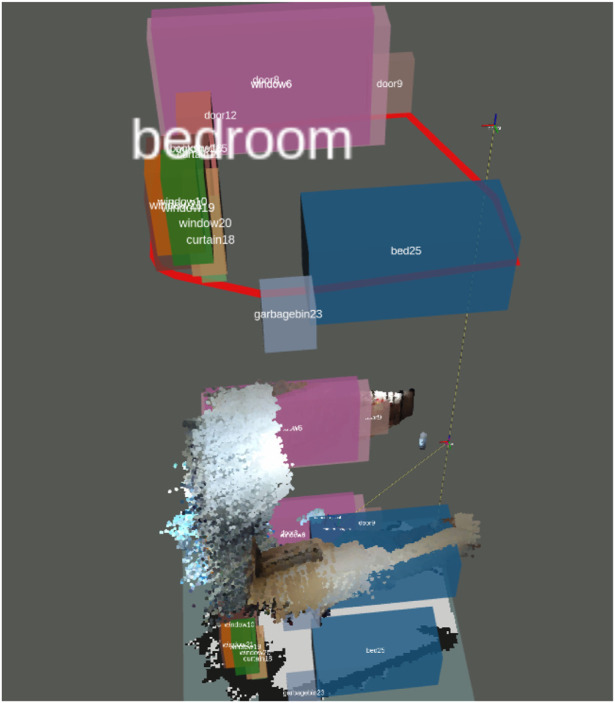
LLMs inferring room type.

A sample illustration for inferring room types is provided in Figure 7.

### 4.4 RoutePlanner experimental phases

#### 4.4.1 Multi-object navigation

This experiment aimed to evaluate the robot’s ability to navigate to a specific target object (e.g., a chair) among multiple identical objects within a complex indoor environment. The environment included various rooms: three bedrooms, two bathrooms, one kitchen, two living rooms, and a study, each populated with identical objects from the same category.

- Independent Variable: The target object category (e.g., chair, bed, table) that the robot was instructed to navigate to.
- Dependent Variables:
  – Number of Prompts: The number of additional prompts required for the robot to successfully locate and navigate to the specific target object when initial attempts were uncertain or failed.
  – Success Rate: The percentage of successful navigation attempts where the robot correctly identified and reached the target object among identical items in the room.
- Controlled Variables:
  – Room Configuration: The layout of the rooms, which included three bedrooms, two bathrooms, one kitchen, two living rooms, and a study, remained consistent across trials.
  – Number of Trials: Each room configuration was tested 10 times to ensure robustness across different scenarios.
  – Identical Objects: All identical objects in each room were maintained in their original positions to minimize variability.

The robot initially received a general command to navigate to a specific target object, such as “Navigate to the chair.” If the robot encountered uncertainty or failed to locate the target chair among multiple identical objects, additional prompts were provided in a step-by-step manner to assist it in accurately identifying and navigating to the desired object.

- Initial Command: The robot received a generic instruction, such as “Navigate to the chair.” This command aimed to test the robot’s ability to identify and navigate to any chair within the room without additional information.
- First-Level Prompt: If the robot showed uncertainty or could not locate the target object, a first-level prompt was given, specifying the type of room where the target chair was located. For example, “Navigate to the chair in the living room.” This level of specificity helped the robot narrow down its search to a particular room.
- Final-Level Prompt: If the robot still faced difficulty in locating the correct chair, a second-level prompt was provided to further specify the chair’s relative position within the room. For instance, “Navigate to the chair next to the table in the living room.” This additional spatial context helped the robot distinguish the target chair from other similar objects in the same room.

This hierarchical approach to prompting allowed for an increasingly precise identification process, reducing ambiguity and helping the robot locate the intended object among multiple identical items in a complex environment.

## 5 Results

### 5.1 EnvNet result

#### 5.1.1 SLAM + FCAF3D object detection

We evaluated the performance of the FCAF3D model in detecting objects in a real home environment using SLAM-generated dense point clouds. The experiment’s mAP was 0.55, closely matching the official AP@0.5 value of 55.2, but notably lower than the AP@0.25 threshold of 69.7. This difference may be due to the limitations in generating dense point clouds by ORB-SLAM2 and the object layouts and lighting conditions in the real-world environment.

**FIGURE 8:**
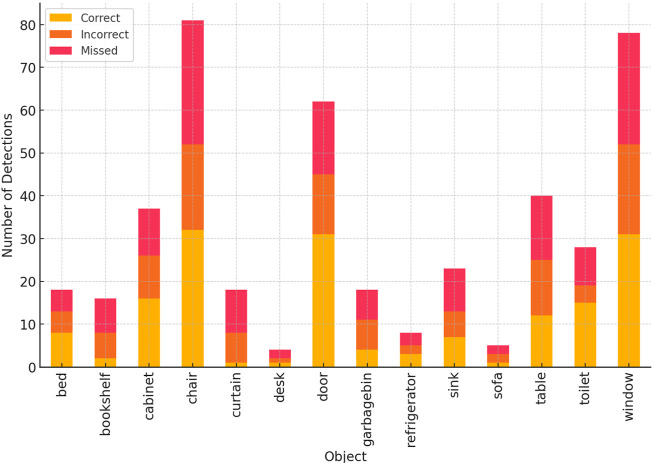
Summary of detection results for different objects.

Figure 8 summarizes the detection performance for different objects, with some objects (e.g., windows and curtains) showing lower detection accuracy due to overlapping features or challenging lighting conditions.

The experimental results indicate that rooms with distinct, easily identifiable objects (e.g., refrigerators in kitchens, beds in bedrooms) were classified with higher accuracy. However, complex or cluttered environments, such as living rooms, presented greater challenges for object detection, leading to reduced classification accuracy.

#### 5.1.2 Error analysis

A deeper analysis of the lower detection accuracy for “windows” and “curtains” (65% and 55% respectively, as shown in Figure 8) reveals two primary causes. 1) Visual Feature Similarity: In RGB images, windows under strong daylight and white curtains often exhibit similar bright, low-texture visual characteristics, leading to frequent confusion between the two categories. 2) Lack of Distinct 3D Features: In the point cloud data, both objects tend to appear as flat, vertical surfaces, lacking the distinctive geometric signatures typical of furniture such as chairs or tables. This makes them inherently challenging for a geometry-focused detector like FCAF3D. These findings expose a key limitation of our current fusion method in handling low-texture, reflective, or co-planar objects, highlighting an important direction for future improvement.

### 5.2 AIBrain result

#### 5.2.1 LLM performance evaluation

**FIGURE 9:**
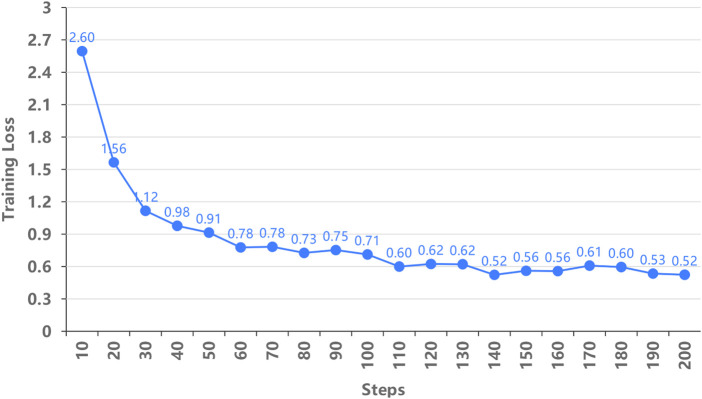
Training loss at different steps.

In this experiment, the Llama 2 model was fine-tuned using LoRA technology with training datasets to improve its ability to process natural language navigation commands. Despite the relatively small dataset, remain the low training loss, as shown in Figure 9, indicating effective learning during the fine-tuning process.

Following fine-tuning, Llama 2 outperformed the other models, particularly when handling simple instructions, achieving 100% accuracy. However, it is important to clarify the context behind this result. The 100% accuracy refers specifically to a set of ten simple instructions that I generated, such as “Navigate to the couch.” These instructions were limited in scope and not derived from a large, diverse dataset. Therefore, while Llama 2 demonstrated perfect accuracy on this small sample, this result should not be interpreted as evidence that it will always achieve perfect understanding universally. It reflects performance on a very controlled subset of tasks, and care must be taken not to overgeneralize from these findings.For more complex tasks, its accuracy remained high at 80%, as shown in Table 3. In contrast, alternative models such as Llama 3 and Gemma had a significant drop in accuracy, particularly with complex instructions.

**TABLE 3:** Accuracy comparison by model and instruction type.

Large language model	Simple instruction	Complex instruction
LLaMA3	80%	60%
LLaMA2(Fine-Tuned)	100%	80%
Gemma	80%	60%
Mistral_q4	60%	50%
Mistral_q6	80%	70%

**FIGURE 10:**
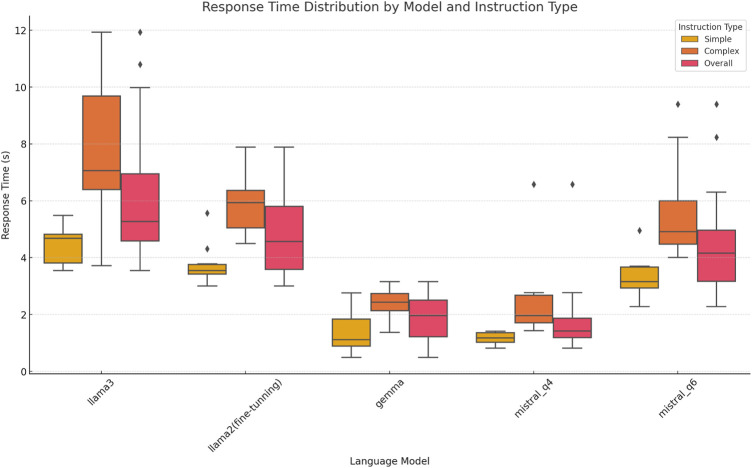
Response time distribution across different models and instruction types.

In terms of response time, Llama 2 again showed a clear advantage, with faster and more stable response times, especially for simple tasks, as illustrated in Figure 10. While response times increased slightly for complex instructions, they remained within an acceptable range.

#### 5.2.2 Room type inference

**FIGURE 11:**
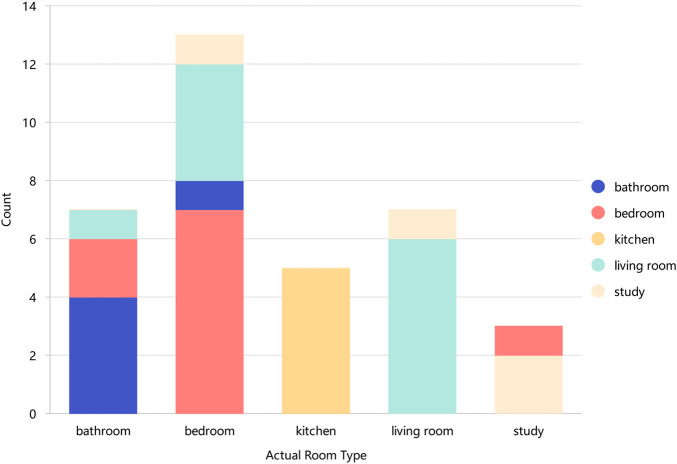
Comparison of actual room types and predicted room types.

In this experiment, room types were inferred using FCAF3D and LLMs based on the detected objects within the room. The classification accuracy in our indoor environment was 65.7%, as shown in Figure 11, which compares actual and predicted room types.

Notably, the confusion observed—such as the misclassification of a “Study” as a “Bedroom”—highlights the challenge of semantic understanding in multi-functional spaces. For example, if a study contains a sofa bed for resting, the presence and visual prominence of the bed may outweigh that of the desk, leading the LLM to an incorrect inference. This demonstrates that a strategy solely reliant on an unweighted list of detected furniture can be brittle in rooms with ambiguous functions. Addressing this limitation would benefit from a more sophisticated prompting strategy that incorporates object spatial relationships and the relative importance of each item.

### 5.3 RoutePlanner result

#### 5.3.1 Multi-object navigation

**FIGURE 12:**
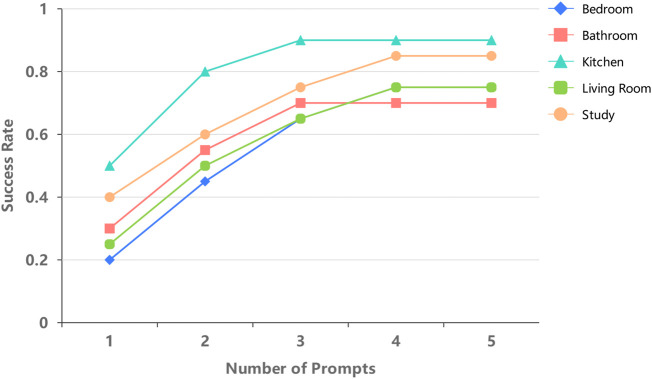
Success rate by room type for various prompts.

The robot’s ability to navigate to a specific target object, such as a chair, among multiple identical objects in various rooms was evaluated. As shown in Figure 12, the success rate of navigation varied depending on the room type and the number of prompts given.

The kitchen demonstrated the highest success rate, with the robot reaching 50% accuracy after the first prompt, quickly rising to 80% by the second, and eventually stabilizing at 90%. This suggests that the kitchen’s straightforward layout enabled more efficient navigation. In contrast, the bedroom proved the most challenging, requiring three prompts to reach a 65% success rate. The presence of multiple bedrooms in the environment likely contributed to the lower performance. These findings suggest that the complexity of object arrangement and room layout significantly impacted the robot’s ability to accurately and efficiently complete navigation tasks.

### 5.4 Qualitative case studies

To complement the quantitative metrics, we present a qualitative analysis of two representative cases to illustrate the framework’s operational dynamics.

#### 5.4.1 Successful complex command execution

A successful instance involved the command: “Check if the window near the sofa is closed.” The process, illustrated in Figure 2, unfolded as follows:

1) AIBrain - Interpretation: The LLM receives the command. Using Chain-of-Thought (CoT) reasoning, it infers that “check if closed” requires visual inspection, identifying the key objects as “window” and “sofa”.
2) AIBrain to EnvNet - Query: It queries EnvNet for the locations of “sofa” and “window”.
3) EnvNet - Localization: EnvNet returns the coordinates of all sofas and windows from its semantic map.
4) AIBrain - Disambiguation & Planning: The AIBrain correlates the locations, identifies the window closest to a sofa, and generates a high-level plan: “Navigate to window at coordinate (x,y).”
5) RoutePlanner - Execution: RoutePlanner computes a path to the target location and executes the navigation.

This case underscores the framework’s strength in decomposing a complex, underspecified command into a coherent sequence of logical queries and physical actions.

#### 5.4.2 Failure case analysis: perception module sensitivity

A failure occurred with the task: “Bring me the apple from the kitchen table.” Analysis indicated the following sequence:

- AIBrain: Successfully interpreted the command “go to kitchen table, get apple.”
- RoutePlanner: Correctly navigated to the kitchen table, enabled by the robot’s pre-built semantic map, which provided the table’s precise location.
- EnvNet (2D Recognition): Upon arrival, the robot’s YOLOv8 module successfully detected the ’apple’ in its 2D camera feed, confirming the target’s presence.
- EnvNet (3D Perception for Interaction): Failure. To proceed with grasping, the robot initiated a real-time, high-resolution scan of the table surface to determine the apple’s exact 3D coordinates. This critical step failed: strong overhead lighting caused severe specular glare on the table, preventing the RGB-D camera from generating a usable point cloud in that region, thereby impeding object localization for manipulation.

Here, the failure did not arise from the decision-making pipeline, but rather from the perception stage. The system’s inability to acquire a reliable 3D representation of the table surface prevented the subsequent (albeit out-of-scope for this work) grasp planning. This highlights the current limitations of the perception module in handling challenging lighting conditions, marking it as a critical area for future robustness improvements.

Together, these qualitative cases demonstrate both the system’s compositional reasoning strengths and its perceptual bottlenecks, providing actionable insights for next-stage development.

## 6 Discussion & future work

This paper introduced a modular AI agent framework designed for home assistance robots, centered on the novel integration of a quantized Large Language Model for device decision-making. Our three-part architecture-comprising EnvNet for perception, AIBrain for reasoning, and RoutePlanner for navigation-demonstrated its viability on resource-constrained hardware. Key results validated our approach: the fine-tuned Llama two model achieved up to 100% accuracy in interpreting simple navigation commands and 80% on complex ones, outperforming several baseline models. Furthermore, our system successfully navigated complex multi-object scenarios with a success rate reaching 90% after hierarchical prompting, indicating a practical solution to environmental ambiguity in home environments.

Despite these promising results, we acknowledge several limitations that define a clear direction for future research. First, the perception module (EnvNet) struggled with objects possessing ambiguous geometric or visual features, such as windows and curtains, leading to a modest mean average precision (mAP) of 0.55 for object detection. Second, our experiments were conducted in a limited number of home environments with a relatively small, synthesized set of user commands. While effective as a proof-of-concept, this scope does not capture the full diversity of real-world scenarios. Finally, our evaluation focused on discrete navigation tasks rather than complex, end-to-end domestic chores, which are essential for validating true long-term autonomy.

Building directly upon these limitations, our future work will proceed along three primary tracks. To enhance perception, we will integrate more advanced multi-modal fusion techniques, such as geometric boundary features, to better distinguish between challenging, co-planar objects. To address the issue of scale and generalizability, we plan a longitudinal study, deploying the system across a large and diverse collection of households to gather a comprehensive dataset of naturalistic interactions. Additionally, we will design a suite of complex, continuous task chains (e.g., “find the dirty cup in the living room, take it to the kitchen, and place it in the sink”) to enable a rigorous evaluation of the system’s end-to-end performance and robustness.

In conclusion, the modular and interpretable AI agent framework proposed in this study offers not only a functional system but also a viable technical blueprint for developing more intelligent, reliable, and safer home assistant robots. By decoupling perception, reasoning, and action, our design provides a transparent alternative to opaque end-to-end models. This emphasis on interpretability and safety is particularly crucial for high-stakes domains—such as elderly care and assistance for individuals with special needs—where trust and reliability are paramount, thereby laying a solid foundation for real-world deployment.

## Data Availability

Publicly available datasets were analyzed in this study. This data can be found here: https://github.com/ScanNet/ScanNet and https://huggingface.co/datasets/haosulab/AI2THOR.
