# Supplementary material for: Integrating large language models for intuitive robot navigation
Source: Front Robot AI. 2025 Sep 4;12:1627937. doi: 10.3389/frobt.2025.1627937 (PMC12444764; doi:10.3389/frobt.2025.1627937)
Supplement: Supplementary file 1 [file Supplementaryfile1.pdf]

Appendix

To clearly demonstrate how our system utilizes LLMs for reasoning and task decomposition, this appendix provides the full templates for the core prompts used in the AIBrain module.

A. Room Classification Prompt

Purpose: To infer the room type based on a list of objects detected by the EnvNet module. The model is instructed to provide a concise, single-word response.

```
[SYSTEM]
You are an expert robot assistant. Your task is to identify the type of a room based on a list of objects detected within it. Respond with only the room name.

[USER]
A robot has scanned a room and detected the following objects:
- sofa
- television
- coffee_table
- remote_control

Based on this list, what is the most likely type of this room?

[ASSISTANT]
living_room
```

Listing 1: Prompt for Room Classification

B. Goal Selection Chain-of-Thought Prompt

Purpose: To interpret a user's ambiguous command and break down the reasoning process using Chain-of-Thought (CoT). The model must first explain its logic step-by-step and then provide the final, structured JSON output for the RoutePlanner.

```
[SYSTEM]
You are an intelligent robot brain. Your task is to interpret a human's command and plan the robot's action. You must first think step-by-step about the user's intention and the most logical place to find the target object. Then, provide the final decision in a structured format: {"room": "room_name", "object": "object_name"}.

[USER]
I'm thirsty, please get me something to drink.

[ASSISTANT]
Here's my thought process:
1.  Analyze the user's request: The user is "thirsty" and wants "something to drink".
2.  Identify potential objects: "Something to drink" could be water, juice, soda, etc. These are usually stored in a refrigerator or found on a kitchen counter.
3.  Identify the most likely location: The most common place to find drinks in a home is the "kitchen". Inside the kitchen, the "refrigerator" is the primary storage location for drinks.
4.  Formulate the plan: The robot should first navigate to the "kitchen". Then, it should locate the "refrigerator" to find a drink.
5.  Final Decision:

{"room": "kitchen", "object": "refrigerator"}
```

Listing 2: Prompt for Goal Selection and CoT Reasoning
